# Potential for Drought Stress Alleviation in Lettuce (*Lactuca sativa* L.) with Humic Substance-Based Biostimulant Applications

**DOI:** 10.3390/plants14152386

**Published:** 2025-08-02

**Authors:** Santiago Atero-Calvo, Francesco Magro, Giacomo Masetti, Eloy Navarro-León, Begoña Blasco, Juan Manuel Ruiz

**Affiliations:** 1 Department of Plant Physiology, Faculty of Sciences, University of Granada, 18071 Granada, Spain; enleon@ugr.es (E.N.-L.); bblasco@ugr.es (B.B.);; 2 Sofbey S.A., Via San Martino 16/A CH-6850, 6850 Mendrisio, Switzerland; fmagro@sipcam.com (F.M.); gmasetti@sofbey.com (G.M.)

**Keywords:** antioxidant system, carboxylation efficiency, chlorophyll fluorescence, fulvic acids, humic acids, plant growth, relative water content, water stress

## Abstract

In the present study, we evaluated the potential use of a humic substance (HS)-based biostimulant in mitigating drought stress in lettuce (*Lactuca sativa* L.) by comparing both root and foliar modes of application. To achieve this, lettuce plants were grown in a growth chamber on a solid substrate composed of vermiculite and perlite (3:1). Plants were exposed to drought conditions (50% of Field Capacity, FC) and 50% FC + HS applied as radicular (‘R’) and foliar (‘F’) at concentrations: R-HS 0.40 and 0.60 mL/L, respectively, and 7.50 and 10.00 mL/L, respectively, along with a control (100% FC). HSs were applied three times at 10-day intervals. Plant growth, nutrient concentration, lipid peroxidation, reactive oxygen species (ROS), and enzymatic and non-enzymatic antioxidants were estimated. Various photosynthetic and chlorophyll fluorescence parameters were also analyzed. The results showed that HS applications alleviated drought stress, increased plant growth, and reduced lipid peroxidation and ROS accumulation. HSs also improved the net photosynthetic rate, carboxylation efficiency, electron transport flux, and water use efficiency. Although foliar HSs showed a greater tendency to enhance shoot growth and photosynthetic capacity, the differences between the application methods were not significant. Hence, in this preliminary work, the HS-based product evaluated in this study demonstrated potential for alleviating drought stress in lettuce plants at the applied doses, regardless of the mode of application. This study highlights HS-based biostimulants as an effective and sustainable tool to improve crop resilience and support sustainable agriculture under climate change. However, further studies under controlled growth chamber conditions are needed to confirm these results before field trials.

## 1. Introduction

Climate change increases different environmental stress conditions that have a detrimental impact on soil properties and crop yields [1,2]. Irrigation is employed for almost 40% of the world’s crop production [3]. This fact, coupled with the critical role of water in essential physiological processes, has made drought one of the most relevant abiotic stresses reducing world agricultural production [4,5,6]. Water stress is strengthened by factors such as light intensity, water holding capacity at the rhizosphere level, high temperature, and low rainfall [3]. In addition, considering that the global population and, consequently, food demand are steadily increasing, discovering new technologies to improve crop tolerance to drought is important [6].

Water deficit affects plant growth at different levels, from the rhizosphere to gas exchange through stomata. In this way, plants exposed to different drought stress conditions usually exhibit lower accumulation of essential nutrients due to a reduced evapotranspiration rate [7]. Additionally, under environmental stressors such as lower water availability, a significant increase in reactive oxygen species (ROS), such as hydroxyl radical (OH^•−^), hydrogen peroxide (H_2_O_2_), and superoxide radical (O_2_^•−^), is typically detected in cells [8,9]. Essential biomolecules such as lipids, proteins, or DNA undergo oxidation by ROS, resulting in DNA mutations, protein denaturalization, lipid peroxidation, and loss of cell membrane stability and cell integrity [10]. In addition, Rubisco CO_2_ assimilation is also affected by water deficiency, reducing photosynthesis performance [11]. All these changes result in reduced plant growth, potentially leading to plant death.

Some strategies shown by plants to reduce drought stress symptoms include increasing root length and root surface area to explore deeper soil layers [5]. Furthermore, plants induce stomatal closure to prevent water loss through transpiration, which significantly decreases CO_2_ fixation and photosynthetic rate and increases ROS accumulation [12]. In this context, improving water use efficiency (WUE) is crucial for maintaining plant growth under drought stress [13]. Another vital aspect is holding ROS at low levels through the antioxidant system. Thus, enzymes such as superoxide dismutase (SOD), catalase (CAT), and ascorbate peroxidase (APX), as well as antioxidants such as glutathione (GSH) and ascorbate (AsA), detoxify ROS, reducing damage to essential biomolecules [14]. Similarly, compatible solutes, such as proline, participate in osmoregulation, thereby avoiding cell dehydration [15,16].

Currently, researchers use different strategies to increase crop tolerance to water deficiency, such as breeding drought-tolerant varieties. However, these techniques require long periods of time to work and are expensive [17]. In this context, the application of bioactive compounds along with nutrient solution (“root” or “radicular” form) or directly sprayed to leaves (“foliar” form) has emerged as a promising tool to enhance crop yield under abiotic conditions [7]. These bioactive molecules are included in the term plant biostimulant (PB), which is defined as molecules that, when applied to plants, enhance growth, abiotic stress tolerance, nutrient uptake and use efficiency, and nutritional quality [18]. Different types of PBs, such as humic substances, are known to improve drought tolerance [19].

Humic substances (HSs) comprise approximately 70% of soil organic matter and are derived from organic matter biodegradation [20]. Three types of HSs have been identified according to their solubility: humin (insoluble at all pH), humic acids (HAs) (soluble only at alkaline pH), and fulvic acids (FAs) (soluble at all pH) [21]. Different sources, such as peat, compost, manure, or lignites (i.e., leonardite), are used for HS extraction and may be employed as PB to increase crop yields and abiotic stress tolerance [7,22]. Nevertheless, HS mechanisms of action to enhance plant abiotic stress tolerance are still a subject of study. Furthermore, the physiological differences between HS application via radicular (together with irrigation) and foliar (directly sprayed to leaves) under abiotic stress conditions have not been thoroughly studied.

Among the different leafy vegetables commonly consumed, lettuce (*Lactuca sativa* L.) is considered the main crop widely cultivated, with a global production of nearly 30 million t per year [23]. The percentage of water in lettuce plants is high (90–95%), and it is considered a sensitive crop to water deficit, where a 50% reduction in FC could drastically reduce plant growth [24]. In a previous growth chamber experiment conducted by our research group, we observed improvements in lettuce growth, photosynthetic capacity, and antioxidant quality following application of a leonardite–HS-based product under optimal growth conditions [25,26]. Based on these findings, we hypothesized that the application of the same HS-based biostimulant, either directly to the roots or to the leaves, could enhance drought tolerance in lettuce by regulating the antioxidant response, water use efficiency (WUE), and photosynthetic activity. Accordingly, this preliminary work aimed to evaluate the efficacy of HS in improving lettuce growth under drought stress and to determine the most effective mode of application (root versus foliar). The findings of this study may provide a basis for further investigations under controlled growth chamber conditions, which are necessary before conducting field trials.

## 2. Results

### 2.1. Shoot and Root Growth, LRWC, and DTI

Drought stress reduced shoot biomass in terms of fresh weight (FW). However, HS applications significantly enhanced shoot FW (*p* < 0.001) compared to drought-stressed plants without biostimulant (Table 1). Similarly, shoot RGR decreased under water-deficit conditions, although it was significantly improved by HSs (*p* < 0.001) compared with the negative control. Comparing between doses, lower shoot growth was observed in lettuces (*Lactuca sativa* L.) treated with R-HS at 0.40 mL/L, whereas higher growth was shown by F-HS at 7.50 mL/L. Moreover, drought stress without HSs significantly reduced LRWC (*p* < 0.001). In contrast, no significant reduction was observed in lettuce treated with HS doses compared to the positive control treatment (100% FC). Water scarcity without biostimulant application decreased root growth (FW, RGR, and surface area). Nevertheless, HSs significantly enhanced root FW and root RGR (*p* < 0.001), indicating that R-HS doses had higher values than control plants. In addition, although no differences were observed in root length, a significant improvement in root surface area was presented in lettuce subjected to HSs (*p* < 0.001), with R-HS 0.60 mL/L and both F-HS doses showing no differences compared to the control treatment. Additionally, DTI was significantly enhanced in lettuce plants subjected to HS applications (*p* < 0.001), with the highest value observed at F-HS 7.50 mL/L (Table 1).

### 2.2. Nutrient Accumulation

K and Ca were reduced under drought stress conditions (*p* < 0.05), whereas Mg was significantly increased (*p* < 0.01) compared to control conditions. However, HS application did not significantly affect K, Ca, or Mg concentrations compared with drought-stressed plants without HSs. Moreover, all HS doses significantly decreased Fe accumulation (*p* < 0.001), being lower in R-HS 0.40 mL/L and F-HS 7.50 mL/L. Additionally, F-HS 10.00 mL/L significantly increased Mn levels. In addition, drought stress decreased Zn concentration (*p* < 0.01), with R-HS 0.60 mL/L being the lowest value (Table 2).

### 2.3. Stress Indicators

**Figure 1:**
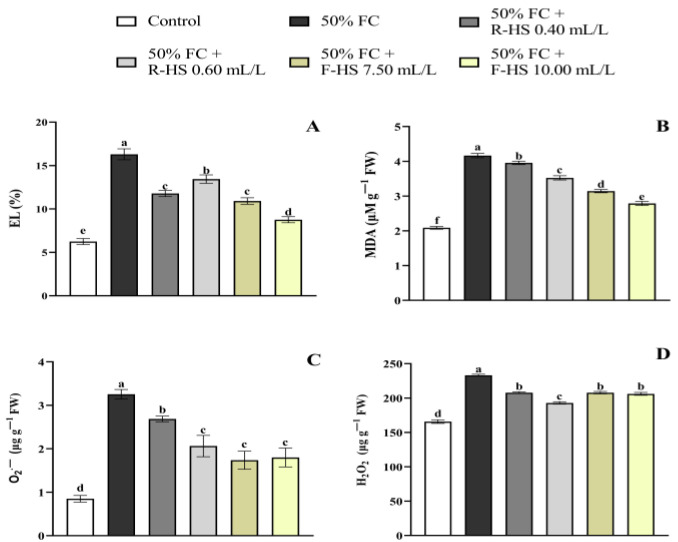
Effect of drought stress and HS application on EL (**A**), MDA (**B**), O_2_^•−^ (**C**), and H_2_O_2_ (**D**) levels. Values are expressed as means ± standard error (*n* = 9). Columns marked with the same letters were not significantly different based on the LSD test (*p* < 0.05).

Water stress without HS application increased the EL. However, plants treated with HSs presented a significant decrease in EL (*p* < 0.001), with F-HS 10.00 mL/L showing the lowest value (Figure 1A). In addition, while MDA accumulation increased under water deficiency, HS application significantly mitigated this effect, with F-HS 10.00 mL/L resulting in the lowest values (*p* < 0.001) (Figure 1B). Under drought conditions, H_2_O_2_ and O_2_^•−^ levels were increased. Nevertheless, the application of HSs significantly reduced O_2_^•−^ accumulation (*p* < 0.001), especially with R-HS (0.40 mL/L) and both F-HS (7.50 and 10 mL/L) (Figure 1C). Additionally, HS application significantly decreased H_2_O_2_ at all doses employed (*p* < 0.001), especially at R-HS 0.60 mL/L (Figure 1D).

### 2.4. Non-Enzymatic and Enzymatic Antioxidants

Drought stress significantly increased total phenol and flavonoid concentrations (*p* < 0.001) compared with control plants. The application of the HS-based product significantly reduced total phenols and flavonoids with respect to stressed plants without biostimulant (*p* < 0.001), except at R-HS 0.40 mL/L for total phenols. In addition, drought (with or without HS) significantly increased total AsA compared with the control treatment (*p* < 0.001), with R-HS 0.60 mL/L showing the highest values and F-HS 7.50 mL/L showing the lowest values. Drought also decreased AsA (*p* < 0.001), except in plants subjected to R-HS 0.60 mL/L, with R-HS 0.40 mL/L showing the lowest concentration. In addition, DHA was significantly increased under water scarcity (*p* < 0.001), being higher in lettuce treated with R-HS and lower with F-HS 7.50 mL/L. Regarding total GSH, drought (without HS) did not affect it compared with the control treatment. All HS doses increased total GSH with respect to the negative control (*p* < 0.001), except R-HS 0.40 mL/L. Additionally, GSH was significantly reduced at R-HS 0.40 mL/L and enhanced at both F-HS, with respect to stressed plants without HS (*p* < 0.001). All HS doses significantly increased GSSG levels (*p* < 0.001), with the highest increase observed at R-HS 0.40 mL/L (Table 3).

**Figure 2:**
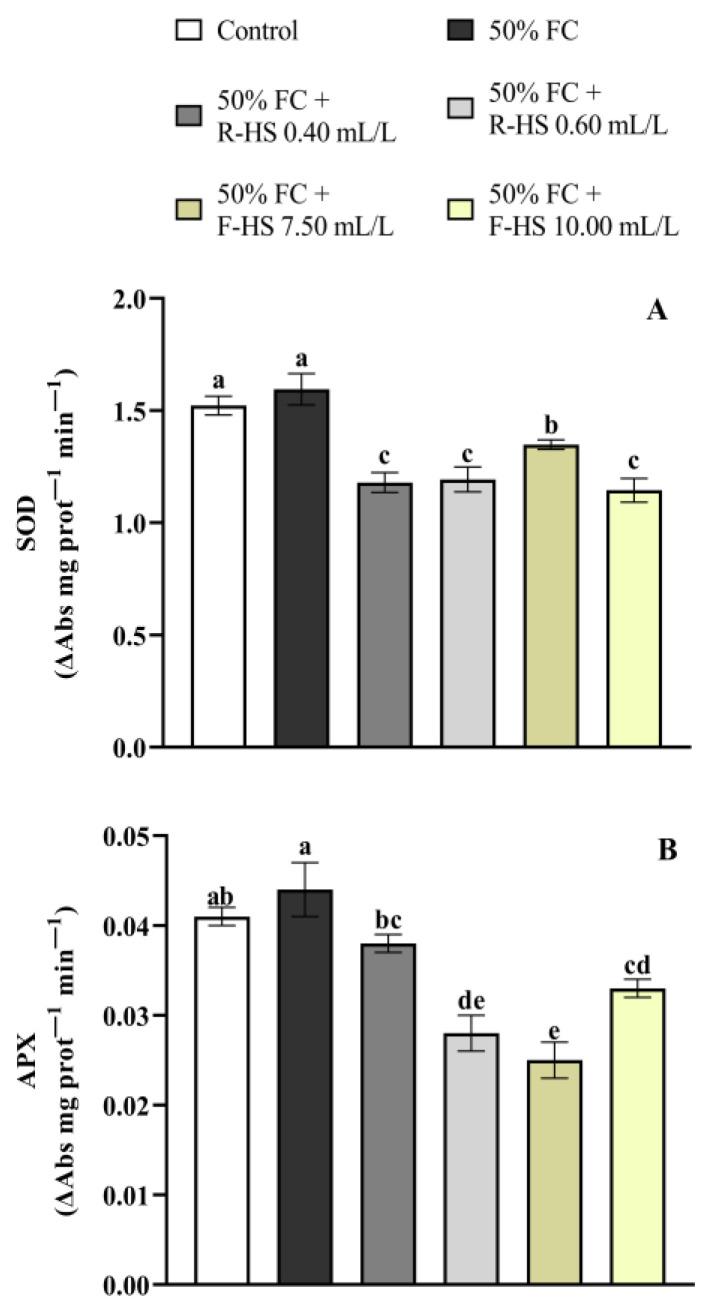
Effect of drought stress and HS application on SOD (**A**) and APX (**B**) activities. Values are expressed as means ± standard error (*n* = 9). Columns marked with the same letters were not significantly different based on the LSD test (*p* < 0.05).

HS applications significantly reduced SOD activity (*p* < 0.001), with F-HS 10.00 mL/L showing the lowest activity (Figure 2A). Similar results were obtained for APX activity, where HS addition significantly decreased it at all doses used (*p* < 0.001), with the lowest activity at 7.50 mL/L F-HS (Figure 2B).

### 2.5. Pro Concentration

**Figure 3:**
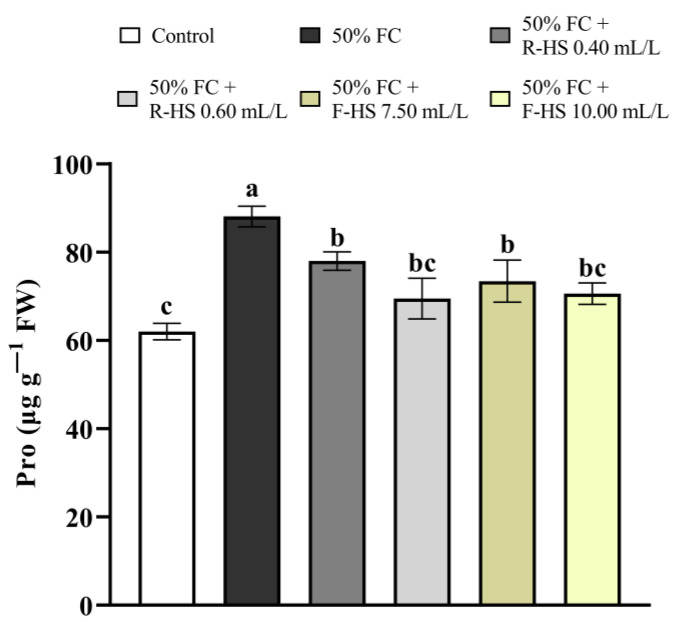
Effect of drought stress and HS application on the concentration of Pro. Values are expressed as means ± standard error (*n* = 9). Columns marked with the same letters were not significantly different based on the LSD test (*p* < 0.05).

Stressed plants without biostimulant, as well as plants treated with 0.40 mL/L R-HS and 7.50 mL/L F-HS, showed a significant increase in Pro concentration (*p* < 0.001) compared with the control. Furthermore, the application of 0.60 mL/L R-HS and 10.00 mL/L F-HS did not significantly affect Pro accumulation compared with the control treatment (Figure 3).

### 2.6. Leaf Gas Exchange Parameters

Drought stress significantly reduced *A* (*p* < 0.001), although this reduction was lower in lettuce plants subjected to HS applications, with F-HS 10.00 mL/L showing the highest *A* values and F-HS 7.50 mL/L the lowest. In addition, the water deficit also diminished *E* compared with the control treatment (*p* < 0.05). A general trend of increased *E* through HS applications, especially with R-HS 0.40 mL/L, was observed with respect to stressed plants without HSs. A significant reduction in *Ci* (*p* < 0.01) was observed in drought conditions (without HSs) and in stressed plants treated with 0.60 mL/L R-HS and 7.50 mL/L F-HS, with respect to the control (100% FC). Furthermore, drought conditions reduced (*p* < 0.001) *gs*, although this reduction was lower in R-HS (0.40 mL/L) and F-HS (10.00 mL/L). Concerning *Vcmax*, it was significantly reduced under drought stress (without HS application) (*p* < 0.001). However, this reduction was less pronounced in plants subjected to HSs, with higher values observed under foliar applications at 7.50 mL/L. Moreover, water scarcity reduced *Jmax* (*p* < 0.001), with this reduction being lower in HS-treated plants and showing root applications at 0.40 mL/L higher values. Finally, WUE was significantly increased with all HS doses (*p* < 0.01), except at R-HS 0.40 mL/L, compared to drought without biostimulant (Table 4).

### 2.7. Chl a Fluorescence

**Figure 4:**
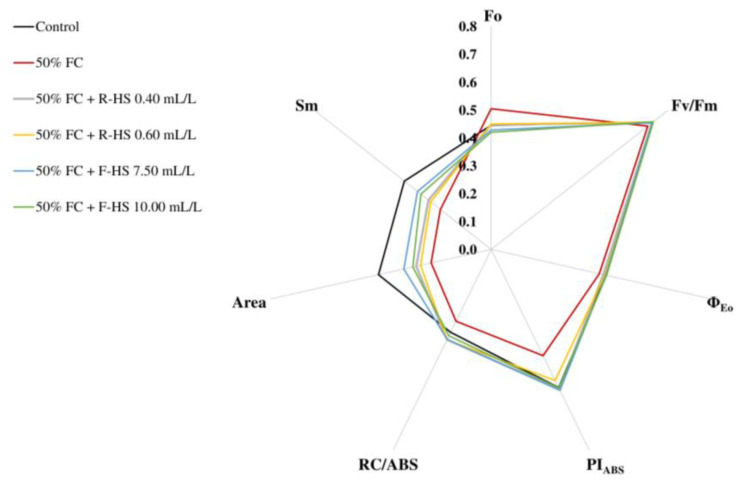
Effect of drought and HS application on initial fluorescence (Fo), maximum quantum yield of primary photochemistry (Fv/Fm), maximum quantum yield of e^−^ transport (Φ_Eo_), performance index (PI_ABS_), proportion of active reaction centers (RC/ABS), area above the fluorescence curve between Fo and Fm (Area), and energy needed to reduce reaction centers (Sm). Values are expressed as means ± standard error (*n* = 6). All results are represented in the same scale, for which the data were normalized.

Although the water deficit without HS application increased the initial fluorescence (Fo), no significant differences were detected with respect to the control treatment. Nevertheless, a significant reduction in Fo was observed in plants supplied with foliar applications at both doses (*p* < 0.05) compared with all other treatments. Furthermore, although drought without HSs decreased Fv/Fm (*p* < 0.001) and Φ_Eo_ (*p* < 0.05), these parameters were not affected in lettuce plants treated with HS doses, except for R-HS 0.60 mL/L, which did not show differences with respect to the negative control. More specifically, foliar applications at 10.00 mL/L offered higher values of Fv/Fm Φ_Eo_. Water stress also reduced PI_ABS_ and RC/ABS (*p* < 0.001). Nevertheless, these reductions were not observed in lettuce subjected to HS, showing higher RC/ABS values under root applications of HS than in control conditions. Similarly, water deficiency decreased Area and Sm (*p* < 0.001), although this decrease was lower in HS-treated plants, especially at foliar doses (Figure 4, Table S1).

### 2.8. Correlation Analysis

**Figure 5:**
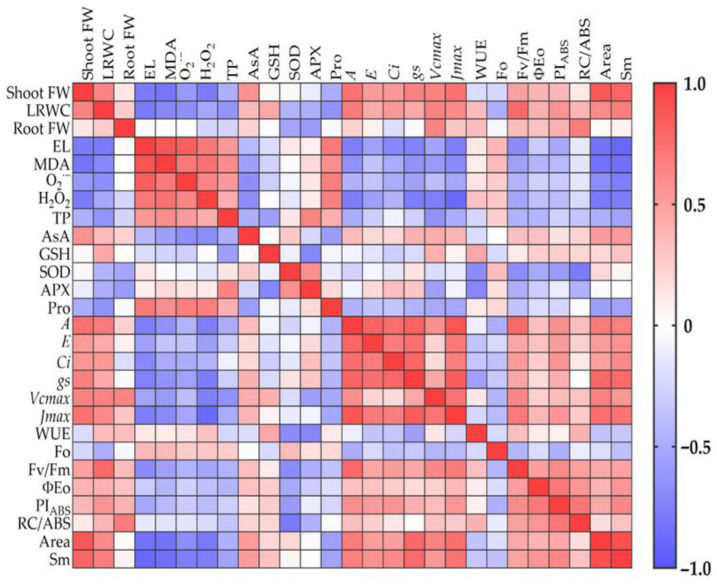
Heatmap matrix showing the correlation among the analyzed physiological parameters. The color of each cell represents the strength and direction of the correlation—red for positive, blue for negative, and light colors for near zero—while the numerical values from −1 to +1 quantify the correlation strength, with values closer to +1 indicating a strong positive correlation and values closer to −1 indicating a strong negative correlation.

A correlation analysis was conducted to explore the relationships among the analyzed physiological indicators. As shown in Figure 5 and Supplementary Table S2, a strong negative correlation was observed between shoot FW and lipid peroxidation (MDA), cell membrane instability (EL), ROS levels (O_2_^•−^ and H_2_O_2_), and Pro accumulation (*p* < 0.001). In contrast, shoot growth showed a significant positive correlation with photosynthetic performance parameters such as *A* (*p* < 0.001), *Vcmax* (*p* < 0.01), *Jmax* (*p* < 0.001), PI_ABS_ (*p* < 0.01), as well as Area and Sm (*p* < 0.001). Although no significant correlation was found between root FW and oxidative damage, root growth showed a positive correlation with certain photosynthetic indicators, including *Vcmax* (*p* < 0.01), PI_ABS_ (*p* < 0.05), and RC/ABS (*p* < 0.001). Concerning photosynthetic performance, *A*, *Vcmax*, and *Jmax* were significantly negatively correlated with MDA (*p* < 0.01) and H_2_O_2_ levels (*p* < 0.001). Chl *a* fluorescence indicators, such as Area and Sm, also exhibited strong negative correlations with EL, MDA, and ROS levels (*p* < 0.001).

## 3. Discussion

Among the different adverse conditions for plant growth derived from anthropogenic activities, water scarcity is one of the main abiotic stresses that reduces crop yields [27]. Plant growth reduction is the first visual symptom observed in plants subjected to water deficiency. The application of biostimulants, together with irrigation water or directly sprayed on leaves, has been presented as an environmentally friendly solution to reduce drought damage [7,16]. Although dry weight is widely recognized as a reliable indicator of drought stress effects, we prioritized fresh weight in the present study due to its agronomic relevance in leafy vegetables, such as lettuce (*Lactuca sativa* L.), which is primarily consumed fresh [28,29].

The root system is the first organ to perceive water scarcity and is involved in water uptake. Thus, improved root growth under drought conditions may enhance substrate exploration for water absorption, thereby promoting growth and mitigating the adverse effects of drought [5]. In our study, the application of different doses of HSs improved root growth in terms of biomass and surface area under drought conditions, especially through root application at 0.60 mL/L. This improved root development facilitated greater water acquisition, as evidenced by the less pronounced reduction in shoot growth in treated plants. A proposed mechanism involves the activation of root H^+^-ATPase by HSs, leading to apoplastic acidification that promotes cell elongation and root growth [30], which may, in turn, sustain shoot development under water stress, although further molecular validation is required. In addition, LRWC is an interesting indicator of leaf hydration status and plant tolerance to drought [31]. While drought reduced LRWC, this reduction was not observed in plants treated with HSs, which could be associated with the improvements in root morphology, as previously reported [32], favouring water uptake and transport. Consequently, the HS-based product improved shoot growth and DTI across all doses and application methods, with the greatest effect observed at a dose of 7.50 mL/L. Similarly, different research works have provided information about the potential of different HS types to enhance drought stress tolerance in various plant species, such as melon (*Cucumis melo* L.) [33], millet (*Setaria italica* Beauv.) [34], tea (*Camellia sinensis* L.) [35], and maize (*Zea mays* L.) [36]. This effect may be attributed not only to the physical modification of the root system for water and nutrient uptake but also to the enhancement of various physiological pathways. Notably, HS applications have been associated with increased photosynthetic efficiency, altered hormonal profiles, such as elevated auxin or abscisic acid levels, enhanced accumulation of compatible solutes, and the attenuation of oxidative damage through reduced ROS levels and lipid peroxidation [35,36].

The accumulation of nutrients in leaves may be reduced under drought conditions [37]. As previously documented, the application of biostimulants, including HSs, could enhance nutrient uptake and translocation by inducing root transporters [34,36]. In our study, the effect of drought stress on nutrient accumulation was observed for K, Ca, and Zn, whereas under HS application, Fe was also reduced. In addition, Mg was increased in drought conditions, and Mn was higher in F-HS 10.00 mL/L. These results suggest that HSs did not consistently enhance nutrient uptake or transporter activity under specific experimental conditions and doses. It could be associated with the fact that HS effects depend on different aspects such as source (coal, peat, lignite), composition (%HA/FA), doses, or rates of application [38].

Under water deficit, the increase in ROS levels causes lipid peroxidation, resulting in increased MDA accumulation, loss of cell membrane stability, and electrolyte leakage [3]. Consequently, plant growth is negatively affected by oxidative damage, as was found in the correlation analysis. Therefore, the maintenance of cell membrane integrity is crucial to maintain a correct cell metabolism under drought stress [16]. In our study, HS treatments reduced lipid peroxidation (MDA levels) and EL, likely due to decreased O_2_^•−^ and H_2_O_2_ levels, particularly with foliar applications. Different studies have reported the potential of HSs to decrease ROS levels and cell membrane damage under adverse growth conditions, conferring stress tolerance. Soil amendments with leonardite–HAs and FA applications decreased H_2_O_2_ and lipid peroxidation in maize plants exposed to drought [39,40]. In this way, HS treatments, particularly via foliar applications, could help maintain cell integrity and sustain cellular metabolism, which is crucial for plant performance under drought conditions.

The antioxidant system contributes to the detoxification of ROS and maintains them at low levels [41,42]. In addition, Pro may also directly detoxify ROS [43], although its main function is related to osmotic adjustments under drought and salinity conditions [44]. Overall, the non-enzymatic and enzymatic antioxidants, as well as Pro accumulation, were lower in lettuce plants subjected to HS treatments, without a clear difference between root and foliar application methods. Although it may seem paradoxical, these results could explain the improvement in lettuce tolerance to drought conditions by HS application. On the one hand, the increased DHA and GSSG levels in HS-treated plants suggest that these plants employ AsA and GSH in ROS detoxification. This indicates that ROS scavenging is maintained through dynamic redox cycling, preventing excessive ROS accumulation without overinvesting in antioxidant synthesis [45,46]. Preserving a balanced redox state without triggering energy-intensive antioxidant pathways indicates that HS might directly regulate ROS production at its origin, possibly by enhancing the efficiency of electron (e^−^) transport chains in chloroplasts, which, in turn, restricts ROS formation [46]. Conversely, the reduction in phenol content, antioxidant enzyme activities, and Pro accumulation after HS applications might reflect an enhanced metabolic efficiency where carbon and nitrogen resources are preferentially allocated to support growth, photosynthesis, and biomass accumulation rather than stress responses [47,48]. This shift can be advantageous under moderate drought, enabling plants to sustain productivity while maintaining cellular integrity. However, future transcriptomic studies are crucial for understanding the role of HS treatments in stabilizing cell membrane integrity and regulating ROS scavenging and antioxidant biosynthesis.

As a typical physiological response, stomatal closure occurs under water stress, reducing the transpiration rate, CO_2_ assimilation capacity by the Rubisco enzyme, and photosynthetic rate [16]. These effects were observed to reduce water loss through transpiration in our experiment [49]. Enhancing the Calvin–Benson cycle and photosynthetic efficiency remains a key objective for improving abiotic stress tolerance [50], given the direct relationship between photosynthesis and plant growth, as was evidenced in the correlation analysis. Furthermore, the improvement of photosynthesis is recognized as one of the main physiological mechanisms of action of HSs [25], primarily through the regulation of the carboxylation efficiency of Rubisco enzymes. Several studies have reported that HS application under water-deficit conditions enhances *A* [51], *gs* [52], and Rubisco efficiency [36], thereby promoting plant growth. In our study, the HS-based product caused a restoration of leaf gas exchange parameters in drought conditions, showing a lower reduction in *gs* and *Ci*, with foliar applications at 10.00 mL/L showing the highest values. Physiologically, these results suggest that HSs may influence stomatal regulation mechanisms, possibly by modulating hormonal signals such as abscisic acid or improving root water uptake capacity, which helps maintain leaf hydration and stomatal aperture [30,32]. As a result, the higher CO_2_ available resulted in the largest *Vcmax* and *A*, compared to drought stress without biostimulant, and particularly with F-HS 10.00 mL/L, indicating higher photosynthetic efficiency that could directly contribute to better plant tolerance [53]. Additionally, the less pronounced decrease in stomatal closure and CO_2_ levels in mesophyll cells could contribute to maintaining the reduction of NADP^−^ to NADPH, thereby explaining the lower ROS levels in HS-treated plants [54].

From leaf gas exchange analysis, an interesting parameter that may also be obtained is *Jmax*, which indicates the maximum rate of e^−^ transport for ribulose phosphate regeneration [55]. The evaluation of *Jmax* is of great interest for understanding the photosynthetic status of plants under different environmental conditions [56]. *Jmax* is decreased under abiotic stress, as has been previously identified [57], which is in line with our results, where drought reduced *Jmax*. Nevertheless, *Jmax* was recovered with HS-based product supplementation. Physiologically, maintaining a higher *Jmax* under drought means that the plant can sustain the photochemical reactions necessary for ATP and NADPH production, which are essential for carbon assimilation and other metabolic processes [58]. This implies that HSs protect or stabilize the photosynthetic apparatus, possibly by maintaining membrane integrity and mitigating oxidative damage to thylakoid membranes and photosystems. By supporting e^−^ transport and ribulose phosphate regeneration, HSs improve the overall efficiency of the Calvin–Benson cycle, ensuring continued carbohydrate production, which is fundamental for energy supply, growth, and stress recovery [15,25]. This capacity may be crucial in drought tolerance, as it enables plants to maintain metabolism and growth when resources are limited [16,36].

In modern agriculture, one of the primary objectives of farmers is to maximize WUE, achieving higher crop productivity with lower irrigation [59,60]. Biostimulants have emerged as an effective strategy for improving WUE and reducing crop yield loss caused by drought [61]. In particular, HSs may enhance WUE, improving water deficit tolerance, as found in maize plants subjected to soil HAs [36]. This result is consistent with those obtained in our study, where root applications at 0.60 mL/L and foliar applications at 7.50 and 10.00 mL/L significantly enhanced WUE. An improvement in the photosynthetic rate contributes to more efficient CO_2_ fixation per unit of transpired water [62]. Additionally, an increase in LRWC indicates better water retention, which is crucial for a higher internal water balance [63]. In addition, the partial maintenance of *gs* under drought with HS applications allows sufficient CO_2_ entry without excessive water loss, optimizing the trade-off between carbon gain and water consumption. Thus, our data indicate that the HS-based biostimulant employed could be a sustainable strategy for retaining more water in plants grown under water scarcity conditions, thereby increasing crop production. However, further research is required in different crops and under field conditions.

In addition to the LICOR 6800, other devices, such as the Handy PEA Fluorimeter, provide valuable information about the functioning of the photosynthetic machinery of plants grown under adverse conditions through the analysis of Chl *a* fluorescence [16,64,65]. In abiotic stresses, such as drought, most of the light absorbed by Chl is not employed in photosynthesis, so it is reflected as fluorescence. Thus, Chl *a* fluorescence increases in plants exposed to abiotic stress, indicating higher Fo values in drought-stressed plants without biostimulants. Furthermore, different Chl *a* fluorescence parameters, such as Fv/Fm, RC/ABS, PI_ABS_, Area, or Sm, are reduced under drought conditions due to photochemical activity imbalances [66], which was observed in our study, especially in plants that were not treated with the biostimulant. However, HS treatments, particularly through foliar applications, enhanced all Chl *a* fluorescence parameters, which could positively affect plant growth, as shown in the correlation analysis. Our results agree with those of van Tol de Castro et al. (2022) [67], who found an improvement in drought tolerance in rice plants through better photosynthetic status measured by emission kinetics of Chl *a* fluorescence, which has also been described in *Brassica napus* L. [68] and in millet seedlings [51]. Therefore, our data suggest that HS applications may contribute to the reduction of photochemical reaction imbalance and protection of the photosynthetic apparatus, which promotes better growth under drought stress [16].

## 4. Materials and Methods

### 4.1. Plant Material and Growing Conditions

Lettuce (*Lactuca sativa* L. cv. Capitata, “Summer Wonder”) was used as the plant material in this study. This cultivar was selected due to its high nutritional quality and widespread global consumption. Additionally, the physiological effects of HS-based biostimulant applications under optimal nutrient conditions (i.e., in the absence of external abiotic stress) have been previously assessed by our research group [25,26]. Lettuce seeds were germinated and grown for 45 days (10 February 2024 to 27 March 2024) in 3 × 3 × 10 cm perlite-filled trays in a polythene tunnel at Saliplant S.L. nursery in Carchuna, Granada, Spain (36°42′00.8″ N, 3°27′10.5″ W). This growth period is the minimum required to obtain an aerial part of approximately 5 cm, ensuring successful transplantation. Subsequently, lettuce seedlings were transferred to a growth chamber in the Department of Plant Physiology (University of Granada, Spain) under environmentally controlled conditions. The environmental conditions for optimal lettuce growth were temperature 25 °C/15 °C (day/night), relative humidity 60–80%, photoperiod of 16 h/8 h, and photosynthetic photon-flux density (PPFD) of 350 μmol m^−2^ s^−1^ (estimated through a sensor SB quantum 190, LI-COR Inc., Lincoln, NE, USA). Lettuce plants were grown in individual pots with a 3:1 vermiculite/perlite (3:1) mixture. Pots (19.5 cm high, 12 cm lower diameter, 15 cm upper diameter, and 4 L volume) were arranged in trays (55 cm × 40 cm × 8.5 cm). During the experiment, a complete nutrient solution was added to the plants every 3 days. The pH of this nutritive solution was adjusted to 5.5–6 at each irrigation using a diluted acid (H_3_PO_4_) or base (KOH). This solution contained 4 mM KNO_3_, 3 mM Ca(NO_3_)_2_·4H_2_O, 1 mM NaH_2_PO_4_·2H_2_O, 1 mM KH_2_PO_4_, 2 mM MgSO_4_·7H_2_O, 0.25 mM CuSO_4_·5H_2_O, 1 µM ZnSO_4_·7H_2_O, 2 µM MnCl_2_·4H_2_O, 0.1 µM Na_2_MoO_4_·2H_2_O, 10 µM HBO_3_, and 5 µM Fe-chelate (Sequestrene; 138FeG100).

### 4.2. Description of Treatments and Experimental Design

Treatments were started 7 days after transplantation and were maintained for 30 days. Treatments consisted of control conditions, where plants were well-watered at 100% FC, plants were treated with drought stress (50% FC), and plants were subjected to drought stress (50% FC) and HS application through two radicular (R-HS) doses (0.40 and 0.60 mL/L) and two foliar (F-HS) doses (7.50 and 10.00 mL/L). Given the high-water content in lettuce leaves (90–95%), a reduction to 50% FC is usually considered sufficient to induce water stress [24]. Additionally, in a previous experiment conducted by our research group in lettuce, a reduction to 75% FC was sufficient to decrease yield and plant performance [16]. Therefore, we selected 50% FC as an appropriate condition to induce water stress in lettuce. HS application was carried out through BLACKJAK^®^, a leonardite-HS-based product provided by Sofbey S.A. (Mendrisio, Switzerland) (https://www.sofbey.com/product/blackjak/ (accessed on 23 March 2021)), whose chemical composition has been previously reported [69]. These doses were selected based on a prior screening under optimal growth conditions, where higher and lower doses did not significantly affect plant growth and physiology [25,26]. For root applications, HSs were dissolved in the nutritive solution, and 100 mL of the solution was applied to the top of each lettuce pot to promote direct contact of the HS with the roots. Foliar applications were performed by dissolving the HS-based biostimulant in distilled water and directly spraying 12.5 mL onto the leaves of each lettuce plant. A total of three applications were performed with a periodicity of 10 days between each. Hence, six treatments were employed, each with three replications (defined as three separate trays per treatment) and eight plants per replication. The experimental design followed a completely randomized design within the growth chamber, where trays were periodically repositioned to minimize positional effects such as light or temperature variation.

### 4.3. Plant Harvesting and Analysis of Growth Parameters

Lettuce plants were harvested 30 days after treatment initiation. All plants from each treatment were cut, washed with distilled water, dried with filter paper, and weighed to obtain the shoot and root FW. For each treatment, leaves from nine plants were homogenized and frozen at −45 °C for biochemical analyses, while leaves from another set of nine plants from the same treatment were oven-dried at 60 °C for nutrient analysis. In addition, the root surface area was quantified using a LI-COR optical reader (LI-3000A, LI-COR Inc., Lincoln, NE, USA), and the length of the longest root was measured using a ruler. Shoots and roots from a subset of 6 plants of a similar size were weighed before starting treatments (FWi = initial fresh weight). In this way, RGR was estimated using the formula proposed by Navarro-León et al. (2023) [41]: RGR = (ln FWf − ln FWi)/(Tf − Ti), where FWf = final FW, Tf = 30, and Ti = 0. The RGR was calculated based on FW, considering that lettuce is a leafy vegetable that accumulates a high amount of water in its leaves and is primarily consumed fresh [25,29]. In addition, to obtain the leaf relative water content (LRWC), leaves from three plants per treatment were harvested, washed, dried with filter paper, and weighed, obtaining the FW from each leaf. These leaves were immersed in water-filled plastic trays for 3 h. Afterwards, leaves were weighed to obtain the turgid weight (TW). Finally, the leaves were dried at 60 °C for 48h and weighed again to obtain the dry weight (DW). The LRWC was calculated as follows: LRWC (%) = [(FW − DW)/(TW − DW)] × 100 [70]. The drought tolerance index (DTI) was also calculated as DTI = (Total FW of stressed plants/Total FW of control plants) × 100, where total FW was the sum of FW of leaves and roots [71].

### 4.4. Determination of Nutrient Concentration

We used 0.15 g of dry leaves from six plants per treatment to determine the concentration of potassium (K), calcium (Ca), magnesium (Mg), iron (Fe), manganese (Mn), and zinc (Zn) through a wet digestion with nitric acid (HNO_3_)/perchloric acid (HClO_4_) (*v*/*v*) and H_2_O_2_ (30%) [72]. The nutrient concentration was measured by ICP-MS (X-Series II; Thermo Fisher Scientific Inc., Waltham, MA, USA).

### 4.5. Determination of Oxidative Stress Indicators

The cell membrane stability was determined by the electrolyte leakage (EL) test [16]. Fresh leaves of plants were cut, weighed (0.3 g), washed with distilled water, and placed in a test tube. A total of 30 mL of distilled water was added to each tube, and the tube was vortexed for 1 min. The initial conductivity (EC1) was measured using a conductivity meter (Cond 8; XS Instruments, Carpi, Italy). Afterwards, the tubes were placed in a water bath (100 °C, 20 min) to extract the remaining electrolytes. After this incubation, the final conductivity (EC2) was determined, and EL was calculated as EL = (EC1/EC2) × 100.

Malondialdehyde (MDA) concentration was quantified following the method described in Fu and Huang (2001) [73]. The absorbance of the supernatants was recorded at 532 and 600 nm to correct turbidity. H_2_O_2_ concentration was colorimetrically determined at 350 nm following the method described by Junglee et al. (2014) [74] using potassium iodine (KI). The O_2_^•−^ concentration was assayed at 580 nm by measuring the reduction of nitroblue tetrazolium (NBT) [75].

### 4.6. Determination of Total Phenol, Flavonoids, AsA, and GSH

The total phenol concentration was measured at 725 nm using the Folin–Ciocâlteu reagent and a curve of caffeic acid, whereas the flavonoid concentration was determined at 415 nm against a rutin curve [76]. Total and reduced AsA were assayed through Fe^3+^ reduction by AsA at 525 nm. Dehydroascorbic acid (DHA) was calculated as total AsA–reduced AsA [77]. Following the DTNB oxidation method, total GSH and oxidized GSH (GSSG) were determined at 412 nm. Reduced GSH was calculated as the total GSH–GSSG ratio [78].

### 4.7. Determination of the SOD and APX Activities

The SOD activity was measured according to the method described in [79], which is based on the inhibition of NBT, whereas the APX activity was assayed by measuring the reduction of H_2_O_2_ for 5 min at 290 nm [80].

### 4.8. Quantification of the Pro Concentration

Proline (Pro) determination was performed following the method described by Irigoyen et al. (1992) [81], with minor modifications. Pro was extracted from 0.1 g of frozen leaves using 83% ethanol. After centrifugation for 10 min at 2800× *g*, the supernatant was incubated for 45 min at 100 °C with a mixture of ninhydrin, distilled water, and acetic acid. The absorbance was measured at 515 nm using a Pro curve.

### 4.9. Photosynthetic Gas Exchange Parameters

The photosynthetic leaf gas exchange parameters were recorded using an infrared gas analyzer (LICOR 6800 Portable Photosynthesis System (IRGA: LICOR Inc., Lincoln, NE, USA). The environmental conditions of the LICOR leaf chamber were as follows: 350 μmol m^−2^ s^−1^ of PAR (photosynthetically active radiation, 70% relative humidity, 400 µmol mol^−1^ CO_2_ concentration, leaf temperature 30 °C, and chamber fan mixing speed 10,000 rpm [16]. A total of nine measurements per plant were taken on intermediate leaves of six similarly sized plants per treatment, corresponding to two plants per replicate. The mean value from these nine measurements was employed for net photosynthetic rate (*A*), transpiration rate (*E*), and intercellular CO_2_ (*Ci*). The WUE was calculated as *A*/*E*. After these measurements, a rapid *A*-*Ci* response curve (RACiR) was run with increasing CO_2_ concentrations from 10 to 510 µmol mol^−1^, and *A* and *Ci* were measured every 2 s for 7 min. To correct the data, a RACiR curve was also run without a leaf. Then, the Rubisco maximum carboxylation rate (*Vcmax*) and the maximum rate of e^−^ transport (*Jmax*) were obtained by the “Plantecophys” package in R [82] (http://www.bitbucket.org/remkoduursma/plantecophys (accessed on 23 March 2021)), adjusting the data according to the Farquhar model [83].

### 4.10. Analysis of Chlorophyll a Fluorescence

After 20 min of adaptation to darkness, chlorophyll (Chl) *a* fluorescence was measured using a Handy PEA Chlorophyll Fluorimeter (Hansatech Ltd., King’s Lynn, Norfolk, UK). Fluorescence was induced with a light intensity (3000 µmol photons m^−2^ s^−1^) of red light (650 nm) and was recorded in intermediate leaves of six plants of similar size per treatment (corresponding to two plants per replicate). The following parameters were determined: initial fluorescence (Fo), maximum quantum yield of primary photochemistry (Fv/Fm, where Fv is variable fluorescence and Fm is maximum fluorescence), maximum quantum yield of electron (e^−^) transport (Φ_Eo_), performance index (PI_ABS_), proportion of active reaction centers (RC/ABS), Area (the area above the fluorescence curve between Fo and Fm), and energy needed to reduce reaction centers (Sm) [64].

### 4.11. Statistical Procedures

In vivo data (gas exchange and Chl *a* fluorescence) were collected from six individual plants, with blocks included as a random effect in the statistical analysis. For biochemical and nutrient analyses, intermediate leaves from nine plants (three per replicate) were pooled by treatment, yielding nine measurements per parameter or six in the case of nutrients. All data were subjected to statistical evaluation using a one-way ANOVA with 95% confidence interval. Differences between treatments were compared using Fisher’s Least Significant Difference (LSD) test at a 95% probability level. Significance levels are represented as * *p* < 0.05, ** *p* < 0.01, *** *p* < 0.001, and NS not significant. Additionally, to examine the relationships among the physiological indicators, Pearson’s correlation analysis was performed, and the results were presented as a heatmap matrix generated using GraphPad Prism 9.3.1.

## 5. Conclusions

**Figure 6:**
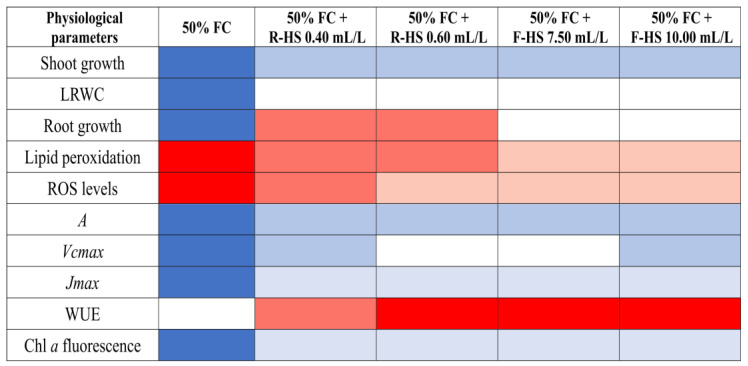
Graphical summary of the effects of drought stress and HS applications on lettuce growth and physiology. The cells filled with blue and red colors represent a negative and positive effect, respectively, compared with the control plants. A more intense coloration reflects a stronger (positive or negative) effect. White cells indicate that the assessed parameter was not affected by the treatment compared with the control plants.

In conclusion, this preliminary study indicates that both root and foliar leonardite–HS-based biostimulant applications show potential for alleviating drought stress in lettuce (*Lactuca sativa* L.) plants by enhancing shoot and root growth, improving photosynthesis capacity and WUE, and reducing oxidative damage (Figure 6). Although greater shoot growth and photosynthetic performance were observed with foliar HS applications, no significant differences were found between the two application methods (root vs. foliar). However, this study does not address the molecular mechanisms underlying the obtained results. Therefore, further molecular studies are required to gain a deeper understanding of the HS-based product’s physiological mechanisms of action in conferring drought stress tolerance in lettuce plants. In addition, further growth chamber experiments are needed to confirm the reproducibility of these effects, which would then justify subsequent field trials.

## Figures and Tables

**Table 1:** Effect of drought and HS application on shoot and root growth, leaf relative water content (LRWC), root length, root surface area, and drought tolerance index (DTI).

	Shoot FW	Shoot RGR	LRWC	Root FW	Root RGR	Root Length	Root Surface Area	DTI
Control	73.03 ± 2.44 a	0.0988 ± 0.0011 a	90.81 ± 0.49 ab	6.03 ± 0.30 c	0.0577 ± 0.0016 c	26.82 ± 0.51	45.58 ± 1.50 a	
50% FC	39.27 ± 0.75 d	0.0782 ± 0.0007 d	85.60 ± 0.13 c	4.91 ± 0.07 d	0.0510 ± 0.0005 d	25.51 ± 1.14	28.14 ± 3.71 c	55.88 ± 1.00 c
50% FC + R-HS 0.40 mL/L	48.08 ± 1.26 c	0.0850 ± 0.0009 c	89.35 ± 0.59 b	6.95 ± 0.16 ab	0.0625 ± 0.0008 ab	26.41 ± 1.70	36.27 ± 3.07 b	69.61 ± 1.70 b
50% FC + R-HS 0.60 mL/L	50.26 ± 2.04 bc	0.0863 ± 0.0013 bc	89.25 ± 0.08 b	7.68 ± 0.33 a	0.0658 ± 0.0014 a	27.52 ± 1.18	42.15 ± 2.24 ab	73.29 ± 2.80 ab
50% FC + F-HS 7.50 mL/L	52.59 ± 0.55 b	0.0880 ± 0.0003 b	90.92 ± 0.82 ab	6.64 ± 0.30 bc	0.0609 ± 0.0015 bc	28.19 ± 1.95	45.22 ± 1.46 a	74.92 ± 0.91 a
50% FC + F-HS 10.00 mL/L	50.66 ± 0.95 bc	0.0862 ± 0.0007 bc	91.37 ± 0.94 a	5.87 ± 0.47 c	0.0565 ± 0.0027 c	26.17 ± 0.70	38.20 ± 3.22 ab	70.54 ± 1.68 ab
*p*-Value	***	***	***	***	***	NS	***	***
LSD_0.05_	4.28	0.0026	1.86	0.88	0.005	3.80	7.81	5.04

Shoot and root FW are expressed as g plant^−1^; shoot and root RGR as g g^−1^ day^−1^; LRWC and DTI as %; root length as cm; root surface area as cm^2^. Values are means ± standard error (*n* = 9). Significance levels are represented as *** (*p* < 0.001) and NS (not significant). Values with different letters indicate significant differences.

**Table 2:** Effect of drought stress and HS application on K, Ca, Mg, Fe, Mn, and Zn concentrations.

	K	Ca	Mg	Fe	Mn	Zn
Control	62.25 ± 2.43 a	9.25 ± 0.31 a	4.92 ± 0.18 b	0.221 ± 0.001 a	0.178 ± 0.006 b	56.14 ± 2.25 a
50% FC	56.25 ± 1.34 b	8.30 ± 0.21 b	6.08 ± 0.16 a	0.230 ± 0.003 a	0.179 ± 0.004 b	50.31 ± 1.15 b
50% FC + R-HS 0.40 mL/L	54.81 ± 1.07 b	7.95 ± 0.18 b	5.75 ± 0.15 a	0.143 ± 0.008 c	0.190 ± 0.005 ab	46.59 ± 0.73 bc
50% FC + R-HS 0.60 mL/L	52.59 ± 0.21 b	8.39 ± 0.05 b	6.03 ± 0.06 a	0.199 ± 0.009 b	0.192 ± 0.002 ab	43.87 ± 0.91 c
50% FC + F-HS 7.50 mL/L	55.77 ± 1.96 b	8.39 ± 0.31 b	5.87 ± 0.26 a	0.163 ± 0.008 c	0.190 ± 0.008 ab	48.07 ± 1.97 bc
50% FC + F-HS 10.00 mL/L	56.47 ± 1.72 b	7.99 ± 0.25 b	6.16 ± 0.21 a	0.195 ± 0.007 b	0.204 ± 0.006 a	49.95 ± 1.16 b
*p*-Value	*	*	**	***	*	**
LSD_0.05_	4.98	0.73	0.55	0.020	0.016	4.53

All nutrients are expressed as mg g^−1^ DW, except for Mn and Zn, which are expressed as µg g^−1^ DW. Values are means ± standard error (*n* = 6). Significance levels are represented as * (*p* < 0.05), ** (*p* < 0.01), and *** (*p* < 0.001). Values with different letters indicate significant differences.

**Table 3:** Effect of drought and HS application on total phenol and flavonoid concentrations and AsA and GSH forms.

	Total Phenols	Flavonoids	Total AsA	AsA	DHA	Total GSH	GSH	GSSG
Control	1.71 ± 0.08 b	1.55 ± 0.03 b	93.31 ± 4.43 d	54.29 ± 1.61 a	39.03 ± 2.82 e	79.01 ± 3.16 cd	45.32 ± 2.90 c	33.69 ± 0.31 c
50% FC	2.54 ± 0.08 a	1.95 ± 0.10 a	118.42 ± 2.41 b	35.05 ± 1.00 c	83.37 ± 1.90 c	76.54 ± 7.33 d	44.35 ± 5.19 c	32.19 ± 2.14 c
50% FC + R-HS 0.40 mL/L	2.52 ± 0.07 a	1.66 ± 0.08 b	124.86 ± 1.59 b	30.00 ± 0.97 d	95.11 ± 1.69 a	75.75 ± 3.31 d	29.22 ± 2.13 d	46.52 ± 1.36 b
50% FC + R-HS 0.60 mL/L	1.72 ± 0.07 b	1.22 ± 0.03 c	141.29 ± 4.73 a	51.56 ± 2.82 a	91.55 ± 2.00 ab	91.13 ± 1.55 c	38.18 ± 1.32 cd	52.95 ± 2.63 a
50% FC + F-HS 7.50 mL/L	1.43 ± 0.05 c	0.96 ± 0.04 d	102.25 ± 1.11 c	40.28 ± 0.48 b	62.41 ± 0.88 d	169.99 ± 5.08 a	125.58 ± 4.43 a	44.41 ± 0.66 b
50% FC + F-HS 10.00 mL/L	1.73 ± 0.09 b	0.99 ± 0.05 d	123.68 ± 1.86 b	37.46 ± 0.58 bc	86.23 ± 1.43 bc	118.22 ± 3.43 b	70.89 ± 2.30 b	47.33 ± 1.45 b
*p*-Value	***	***	***	***	***	***	***	***
LSD_0.05_	0.21	0.18	8.62	4.20	5.36	12.62	9.62	4.71

Total phenols and flavonoids are expressed as mg g^−1^ FW, and the AsA and GSH forms are expressed as µg g^−1^ FW. Values are means ± standard error (*n* = 9). Significance levels are represented as *** (*p* < 0.001) and NS (not significant). Values with different letters indicate significant differences.

**Table 4:** Effect of drought stress and HS application on leaf gas exchange parameters.

	*A*	*E*	*Ci*	*gs*	*Vcmax*	*Jmax*	WUE
Control	9.35 ± 0.92 a	3.58 ± 1.07 a	359.55 ± 10.42 a	0.148 ± 0.022 a	44.21 ± 3.14 a	101.88 ± 1.87 a	2.29 ± 0.15 c
50% FC	4.64 ± 0.05 c	1.59 ± 0.16 b	275.48 ± 10.01 c	0.039 ± 0.002 c	24.13 ± 0.98 c	43.05 ± 0.10 c	2.61 ± 0.06 bc
50% FC + R-HS 0.40 mL/L	7.61 ± 0.26 b	2.93 ± 0.41 ab	325.11 ± 19.02 ab	0.078 ± 0.010 b	36.69 ± 1.87 b	76.67 ± 4.32 b	2.95 ± 0.24 ab
50% FC + R-HS 0.60 mL/L	7.63 ± 0.28 b	2.25 ± 0.13 ab	292.84 ± 12.94 bc	0.063 ± 0.004 bc	42.16 ± 1.41 a	74.79 ± 3.30 b	3.27 ± 0.04 a
50% FC + F-HS 7.50 mL/L	6.74 ± 0.47 b	2.13 ± 0.10 ab	272.65 ± 10.10 c	0.055 ± 0.003 bc	45.90 ± 0.09 a	75.42 ± 1.62 b	3.28 ± 0.19 a
50% FC + F-HS 10.00 mL/L	7.69 ± 0.65 b	2.61 ± 0.26 ab	345.13 ± 12.70 a	0.077 ± 0.008 b	33.39 ± 1.09 b	73.07 ± 6.20 b	3.27 ± 0.08 a
*p*-Value	***	*	**	***	***	***	**
LSD_0.05_	1.61	1.51	39.67	0.033	5.26	18.29	0.45

*A* is expressed as µmol m^−2^ s^−1^; *E* is expressed as mmol m^−2^ s^−1^; *Ci* is expressed as µmol mol^−1^; *gs* is expressed as mol m^−2^ s^−1^; *Vcmax* and *Jmax* are expressed as µmol m^−2^ s^−1^. Values are means ± standard error (*n* = 6). Significance levels are represented as * (*p* < 0.05), ** (*p* < 0.01), and *** (*p* < 0.001). Values with different letters indicate significant differences.

## Data Availability

The data underlying this article will be shared upon reasonable request to the corresponding author.

## Supplementary Materials

The following supporting information can be downloaded at https://www.mdpi.com/article/10.3390/plants14152386/s1, Table S1: Effect of water deficit and HS application on Fo, Fv/Fm, Φ_Eo_, PI_ABS_, RC/ABS, Area, and Sm. Table S2: Pearson correlation coefficients among the analyzed parameters.

## Abbreviations

The following abbreviations are used in this manuscript:

*A*	Net photosynthetic rate
APX	Ascorbate peroxidase
AsA	Ascorbate
*Ci*	Intercellular CO_2_
DHA	Dehydroascorbate
DTI	Drought tolerance index
*E*	Transpiration rate
EL	Electrolyte leakage
Φ_Eo_	Maximum quantum yield of electron transport
Fo	Initial fluorescence
Fv/Fm	Maximum quantum yield of primary photochemistry
*gs*	Stomatal conductance
GSH	Glutathione
GSSG	Oxidized glutathione
*Jmax*	Maximum rate of electron transport
LRWC	Leaf relative water content
MDA	Malondialdehyde
PI_ABS_	Performance index
Pro	Proline
RC/ABS	Proportion of active reaction centers
Sm	Energy needed to reduce reaction centers
SOD	Superoxide dismutase
*Vcmax*	Rubisco maximum carboxylation rate
WUE	Water use efficiency
